# Camrelizumab Plus Apatinib in Treatment-Naive Patients With Advanced Nonsquamous NSCLC: A Multicenter, Open-Label, Single-Arm, Phase 2 Trial

**DOI:** 10.1016/j.jtocrr.2022.100312

**Published:** 2022-03-30

**Authors:** Shengxiang Ren, Jianxing He, Yong Fang, Gongyan Chen, Zhiyong Ma, Jianhua Chen, Renhua Guo, Xiaoyan Lin, Yu Yao, Gang Wu, Quanren Wang, Caicun Zhou

**Affiliations:** a Department of Medical Oncology, Shanghai Pulmonary Hospital, Tongji University, Shanghai, People’s Republic of China; b Department of Thoracic Surgery, First Affiliated Hospital of Guangzhou Medical University, Guangzhou, People’s Republic of China; c Department of Oncology, Sir Run Shaw Hospital, Zhejiang University School of Medicine, Hangzhou, People’s Republic of China; d Department of Oncology, Harbin Medical University Cancer Hospital, Harbin, People’s Republic of China; e Department of Oncology, Henan Cancer Hospital, Zhengzhou, People’s Republic of China; f Department of Medical Oncology, Hunan Cancer Hospital, Changsha, People’s Republic of China; g Department of Oncology, Jiangsu Province Hospital, Nanjing, People’s Republic of China; h Department of Oncology, Fujian Medical University Union Hospital, Fuzhou, People’s Republic of China; i Department of Medical Oncology, The First Affiliated Hospital of Xi’an Jiaotong University, Xi’an, People’s Republic of China; j Department of Thoracic Oncology, Union Hospital affiliated to Tongji Medical College of Huazhong University of Science and Technology, Wuhan, People’s Republic of China; k Department of Clinical Development, Jiangsu Hengrui Pharmaceuticals Co., Ltd., Shanghai, People’s Republic of China

**Keywords:** Immunotherapy, PD-1, VEGFR, lung nonsquamous cell carcinoma

## Abstract

**Introduction:**

Our preclinical work suggests that low-dose angiogenesis inhibition could potentiate programmed cell death protein 1 and programmed death-ligand 1 (PD-L1) blockade. In a cohort of our multicenter phase 1b and 2 study (NCT03083041), promising antitumor activity was observed with camrelizumab plus low-dose apatinib in chemotherapy-pretreated patients with advanced nonsquamous NSCLC. We hereby reported the results in treatment-naive patients (cohort 4) from the same study.

**Methods:**

Eligible patients had untreated advanced nonsquamous NSCLC with a high tumor mutational burden (TMB) (tissue TMB >10 mutations per megabase or blood TMB ≥1.54 mutations per megabase) and without sensitizing *EGFR* or *ALK* alterations. Patients received camrelizumab 200 mg intravenously every 2 weeks plus apatinib 250 mg orally once daily. The primary end point was the objective response rate (ORR) per investigator.

**Results:**

A total of 25 patients were enrolled and treated. A total of 10 (40.0%) confirmed partial responses and 13 (52.0%) stable diseases were observed. The ORR was 40.0% (95% confidence interval [CI]: 21.1–61.3) and disease control rate was 92.0% (95% CI: 74.0–99.0). With a median follow-up of 19.5 months, the median progression-free survival was 9.6 months (95% CI: 5.5–not reached), whereas the overall survival was not reached; the median duration of response was 15.6 months (95% CI: 3.8–not reached). Similar ORR and progression-free survival were observed regardless of PD-L1 tumor proportion score (≥1% versus <1%). The most common treatment-related grade 3 or higher adverse events were increased gamma-glutamyltransferase (24.0%), increased alanine aminotransferase (16.0%), and abnormal hepatic function (16.0%).

**Conclusions:**

Frontline camrelizumab plus low-dose apatinib exhibited promising clinical activity with acceptable safety in patients with advanced nonsquamous NSCLC regardless of PD-L1 expression.

## Introduction

Immunotherapies, represented by immune checkpoint inhibitors (ICIs) targeting programmed cell death protein 1 (PD-1) or programmed death-ligand 1 (PD-L1), have revolutionized the treatment of advanced NSCLC and became the cornerstone of first-line therapy for tumors without a targetable driver alteration.^1^ Pembrolizumab, atezolizumab, and cemiplimab have been approved by the U.S. Food and Drug Administration as monotherapy for first-line treatment for patients with advanced NSCLC and high levels of tumor cell PD-L1 expression (∼20% of all cases),^2^, ^3^, ^4^ whereas pembrolizumab, atezolizumab, or nivolumab plus ipilimumab in combination with chemotherapy have been approved for patients with any level of PD-L1 expression.^5^, ^6^, ^7^, ^8^, ^9^ Despite the improved response rates and, thereby, the expansion in the target patient population with the addition of chemotherapy, treatment tolerability and patient quality of life were impacted.^5^, ^6^, ^7^, ^8^ There remains a need for effective chemotherapy-free (chemo-free) combination regimens for the treatment of advanced NSCLC, particularly in the frontline setting, to improve patient compliance and maintain the quality of life.

Immunosuppressive tumor microenvironment, which is characterized by the limited presence or lack of immune-cell infiltration, is an important factor that compromises the efficacy of immunotherapy. A preclinical study found that antiangiogenic therapies targeting the vascular endothelial growth factor (VEGF)-dependent signaling pathway promoted alleviation of hypoxia, efficient tumor infiltration by CD8-positive T cells, and reduced recruitment of tumor-associated macrophages.^10^, ^11^, ^12^, ^13^, ^14^ Therefore, the addition of an antiangiogenic drug to ICI may enhance antitumor immune responses by normalizing the tumor microenvironment. To date, four chemo-free combinations of ICIs and antiangiogenic agents have been approved by the Food and Drug Administration for the treatment of renal cell carcinoma, hepatocellular carcinoma, and endometrial carcinoma.^15^, ^16^, ^17^, ^18^ In advanced NSCLC, sintilimab plus anlotinib as first-line therapy and ramucirumab or lenvatinib plus pembrolizumab as a later-line therapy have exhibited potential in preliminary clinical trials.^19^, ^20^, ^21^

Apatinib, an oral tyrosine kinase inhibitor (TKI) targeting the VEGF receptor-2, has been approved for pretreated advanced gastric cancer and hepatocellular carcinoma in the People's Republic of China.^22^^,^^23^ Camrelizumab, an anti–PD-1 monoclonal antibody, as monotherapy, has exhibited marked efficacy across multiple solid tumors^24^, ^25^, ^26^, ^27^ and has recently been approved in combination with chemotherapy as a first-line treatment for advanced nonsquamous NSCLC without *EGFR* or *ALK* alterations in People's Republic of China.^28^ On the basis of our preclinical findings that low-dose apatinib optimizes tumor microenvironment and potentiates antitumor effect of PD-1 and PD-L1 blockade in lung cancer, we conducted a multicohort, phase 1b and 2 study to evaluate the chemo-free combination of camrelizumab and apatinib.^29^ In a subgroup cohort of chemotherapy-pretreated patients with nonsquamous NSCLC, apatinib at 250 mg once daily (established recommended phase 2 dose) plus camrelizumab was well-tolerated with promising antitumor efficacy.^30^ In addition, in our exploratory analysis, blood tumor mutational burden (bTMB) was found to be associated with tumor response.^31^ Here, we report the efficacy and safety of camrelizumab plus apatinib in a cohort of treatment-naive patients with nonsquamous NSCLC and a high TMB from the same study.

## Materials and Methods

### Study Design and Patients

This is a multicenter, multicohort, open-label phase 1b and 2 study of camrelizumab plus apatinib in NSCLC undertaken in the People's Republic of China. The study protocol was in compliance with the principles of the Declaration of Helsinki and the Good Clinical Practice guidelines and was approved by the ethics committee of each participating center. All patients provided written informed consent before enrolment. The overall design has been previously published (Supplementary Fig. 1).^29^ Briefly, the study initially planned to evaluate camrelizumab plus apatinib in three treated (but naive to immunotherapy) patient cohorts with advanced NSCLC (cohort 1, chemotherapy-pretreated nonsquamous NSCLC and without *EGFR* or *ALK* mutations; cohort 2, chemotherapy or targeted therapy–pretreated NSCLC with *EGFR* or *ALK* mutation; cohort 3, chemotherapy-pretreated squamous NSCLC) in the phase 2 stage, with patient enrolment starting at March 2017. Because of the encouraging efficacy and safety profile observed in patients in cohort 1 (particularly in those with high bTMB), cohort 4 was subsequently added in December 2018 to further evaluate the combination therapy in treatment-naive patients with advanced nonsquamous NSCLC and a high TMB (see Supplementary Data for protocol and amendments). The present article reported the results of cohort 4.

Specifically, eligible patients were aged 18 to 70 years, had pathologically confirmed stage IIIB to IV nonsquamous NSCLC with wild-type *EGFR* and *ALK* and high TMB (as assessed by a central laboratory), had not received previous systemic therapy for advanced disease, at least one measurable lesion per Response Evaluation Criteria in Solid Tumors (RECIST) version 1.1, and with provision of an archival or fresh tumor sample for PD-L1 analysis and adequate organ functions. High TMB was defined as tumor TMB (tTMB) greater than 10 mutations per megabase (muts/Mb)^32^^,^^33^ or bTMB of at least 1.54 muts/Mb on the basis of receiver operating characteristic analysis (Supplementary Fig. 2).^31^ Key exclusion criteria were active or a history of autoimmune disease, untreated central nervous system metastases, radiographic evidence of tumor invasion of a major blood vessel or an unclear boundary with a blood vessel, cavitation in lung lesions with bleeding risks (per investigator's judgment), and previous treatment with PD-1 or PD-L1 inhibitors or apatinib.

### Procedure

All enrolled patients received camrelizumab 200 mg by means of intravenous infusion every 2 weeks plus apatinib 250 mg orally once daily until disease progression, intolerable toxicity, patient withdrawal, or investigator decision. The maximum duration of exposure for camrelizumab was 2 years. Treatment beyond disease progression was allowed when clinical benefits were perceived by the investigator. For the management of adverse events (AEs), the dose of camrelizumab could be delayed (up to 12 wk), but not reduced. Dose delay reduced dosing frequency (first to 5-d on and 2-d off, then to 1-d on and 1-d off) and dose discontinuation was permitted for apatinib.

### End Points and Assessment

The primary end point was objective response rate (ORR) (defined as the proportion of patients with a best overall response of complete response [CR] or partial response [PR]) as assessed by the investigator according to RECIST version 1.1. Secondary end points included disease control rate (DCR) (defined as the proportion of patients with the best overall response of CR, PR, or stable disease), clinical benefit rate (defined as the proportion of patients with CR, PR, or stable disease for ≥24 wk), duration of response (DoR), time to objective tumor response, progression-free survival (PFS), overall survival (OS), 12-month survival rate, and safety. The exploratory analysis included the relationship between biomarker (tumor PD-L1 expression) and efficacy.

Tumor evaluation was performed every 8 weeks in the first 6 months and every 12 weeks thereafter using computed tomography or magnetic resonance imaging until disease progression or initiation of new antitumor treatment. Tumor response was assessed by the investigators according to RECIST version 1.1. PR and CR were required to be confirmed with a subsequent scan at least 4 weeks after the first documentation of the response. Safety was assessed with AEs and was monitored from the time of informed consent to 30 days after the last dose of study treatment. AEs were graded according to National Cancer Institute Common Terminology Criteria for Adverse Events version 4.03. For biomarker analysis, PD-L1 expression was measured using the IHC 22C3 pharmDx kit (Agilent Technologies, Santa Clara, CA); PD-L1-positive was defined as a tumor proportion score of greater than or equal to 1%. TMB was measured in peripheral blood (i.e., liquid biopsy) or tissue (pretreatment tumor biopsy or archival tissue) samples using the BGI Oseq pancancer panel (BGI, Shenzhen, People’s Republic of China) (covers 636 genes and 1.95Mb) on the MGISEQ-2000 platform (MGI Tech Co. Ltd., Shenzhen, People’s Republic of China). TMB was calculated by the number of nonsynonymous mutations (including coding base substitution and indels per megabase) with an allele frequency of greater than or equal to 1% after removing germline polymorphisms and known or predicted driver mutations.

### Statistical Analysis

On the basis of the results of cohort 1 from our study^31^ and trials of nivolumab plus ipilimumab,^32^^,^^33^ the estimated ORR for patients with advanced NSCLC and a high TMB was 50%. With a sample size of 20 patients, the two-sided 90% Clopper-Pearson exact confidence interval (CI) for the ORR would be at most 40% wide. With an ORR of 50%, the lower bound of the exact 90% CI would exclude 30%. Efficacy was analyzed in the full analysis set, including all enrolled patients who received at least one dose of study medication. The safety analysis included all patients who received at least one dose of study medication and had at least one posttreatment safety evaluation record. The 95% CIs of ORR, DCR, and clinical benefit rate were calculated using the Clopper-Pearson method. Median DoR, PFS, and OS were estimated using the Kaplan–Meier method, and the 95% CIs were calculated with the Brookmeyer-Crowley method. OS rates at different time points were estimated using the Kaplan–Meier method, and the 95% CIs were derived using the log-log transformation with back transformation to a CI on the untransformed scale. All statistical analysis was performed using Statistical Analysis System (SAS Institute, Cary, NC) version 9.2 or above.

## Results

### Patient Deposition and Baseline Characteristics

**Figure 1:**
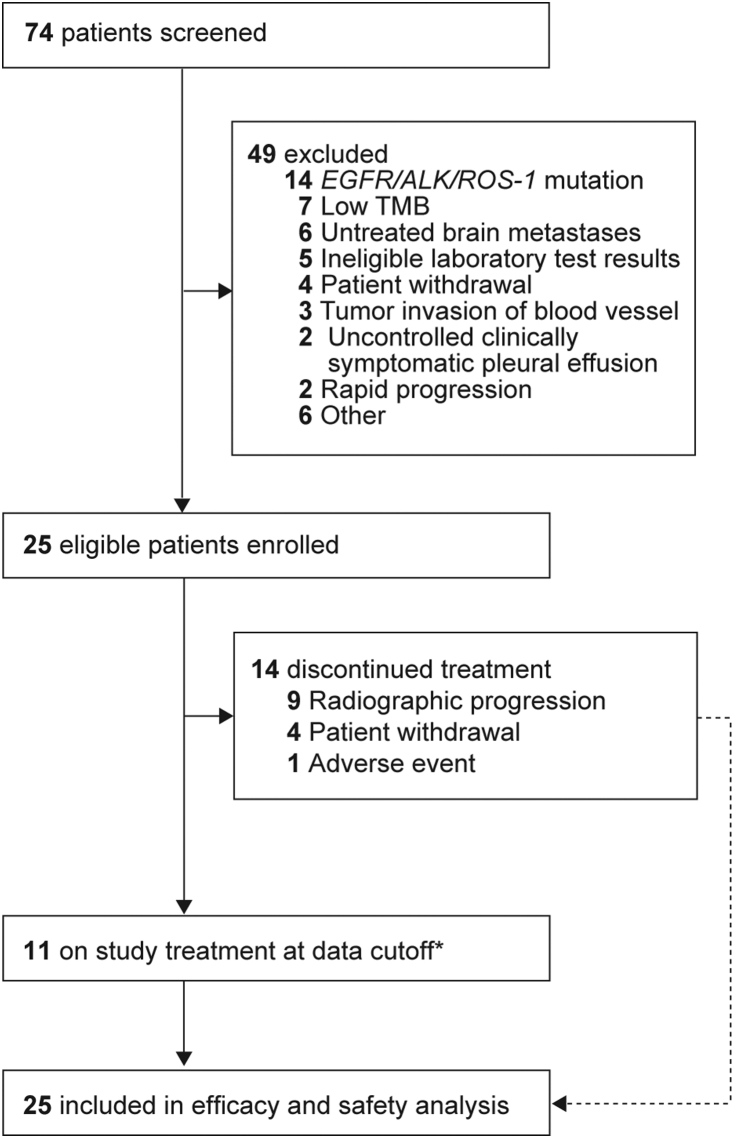
Study profile. ∗Including five patients who were treated beyond the first RECIST version 1.1–defined progression. RECIST, Response Evaluation Criteria in Solid Tumors; TMB, tumor mutational burden.

Between Mar 8, 2019 and Dec 12, 2019, 74 patients were screened and 25 were enrolled, with 7 (9.5%) excluded because of low TMB (Fig. 1). All enrolled patients were treated and included in the efficacy and safety analysis. As of the data cutoff of Jun 12, 2021, the median follow-up was 19.5 months (range: 2.0–26.2). There were 11 patients (44.0%) who remained on study treatment, including 5 (20.0%) patients who were treated beyond the first RECIST version 1.1–defined disease progression (Fig. 1). The reasons for treatment discontinuation were disease progression (nine patients [36.0%]), patient withdrawal (4 [16.0%]), and AE (1 [4.0%]). Subsequently, 10 (40.0%) patients received at least one antitumor therapy including chemotherapy (eight patients [32.0%]) and other PD-1 inhibitors plus an antiangiogenic agent combination (two patients [8.0%]).

Their baseline characteristics are detailed in Table 1. Most patients were men (76.0%), smoker (76.0%), and had stage IV disease (84.0%). All patients had high TMB on the basis of bTMB, with a median bTMB of 3.08 (range: 1.54–13.33) (Supplementary Table 1). There were 60.0% of patients who were PD-L1 expression–positive, and 16.0% had a PD-L1 tumor proportion score of at least 50%.

**Table 1:** Baseline Characteristics

Characteristic	Patients (*N* = 25)
Age, y	
Median (range)	61 (47–69)
Sex, *n* (%)	
Male	19 (76.0)
Female	6 (24.0)
ECOG performance status, *n* (%)	
0	5 (20.0)
1	20 (80.0)
History of smoking, *n* (%)	
Never smoked	6 (24.0)
Current or former smoker	19 (76.0)
Histologic type, *n* (%)	
Adenocarcinoma	23 (92.0)
Large cell carcinoma	1 (4.0)
Others	1 (4.0)
Disease stage, n (%)	
IIIB-C	3 (12.0)
IV	21 (84.0)
Unknown	1 (4.0)
No. of organs with metastasis, *n* (%)	
0	4 (16.0)
1-2	16 (64.0)
>2	5 (20.0)
Median bTMB (range), muts/Mb^a^	3.08 (1.54–13.33)
PD-L1 TPS, *n* (%)	
<1%	10 (40.0)
≥1%	15 (60.0)
≥50%	4 (16.0)

bTMB, blood tumor mutational burden; ECOG, Eastern Cooperative Oncology Group; muts/Mb, mutations per megabase; PD-L1, programmed death-ligand 1; TMB, tumor mutational burden; TPS, tumor proportion score.

a Tissue TMB was assessed in six patients, with a median of 3.08 muts/Mb (range: 1.03–6.15).

### Efficacy

**Figure 2:**
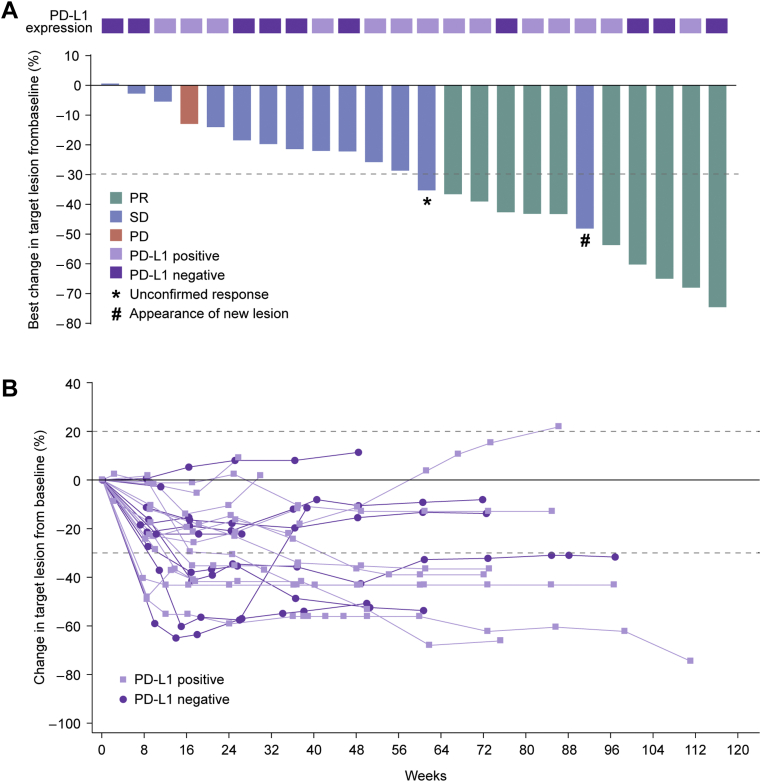
Tumor response. (*A*) The maximum change in target lesion size from baseline. (*B*) Change in target lesion size from baseline over time. Tumor response was assessed by the investigators per RECIST version 1.1. PD, progressive disease; PD-L1, programmed death-ligand 1; PR, partial response; RECIST, Response Evaluation Criteria in Solid Tumors; SD, stable disease.

The best change in target lesion size from baseline is illustrated in Figure 2*A*; 23 of 25 (92.0%) patients experienced a reduction in tumor size. A total of 10 (40.0%) patients achieved a confirmed objective response per the investigator’s assessment and the ORR was 40.0% (95% CI: 21.1–61.3) (Table 2). In addition, one patient had an unconfirmed PR. There were 13 (52.0%) patients who had stable disease and the DCR was 92.0% (95% CI: 74.0–99.0). Among the 10 responders, the median DoR was 15.6 months (95% CI: 3.8–not reached); five (20%) responses were still ongoing at the time of data cutoff (Fig. 2*B*). Subgroup analysis revealed that the ORR was 40.0% (95% CI: 16.3–67.7) in patients with PD-L1–positive expression, as compared with 40.0% (95% CI: 12.2–73.8) in those with PD-L1–negative expression (Table 2).

**Table 2:** Investigator-Assessed Tumor Response

Efficacy Variable	Patients (*N* = 25)
Best overall response, *n* (%)	
Complete response	0
Partial response	10 (40.0)
Stable disease	13 (52.0)
Progressive disease	1 (4.0)
Not evaluable	1 (4.0)
ORR, *n* (%, 95% CI)	
Overall	10 (40.0; 21.1–61.3)
PD-L1 positive	6 (40.0; 16.3–67.7)
PD-L1 negative	4 (40.0; 12.2–73.8)
Median DoR (range), mo	15.6 (3.8–NR)
DCR, *n* (%, 95% CI)	23 (92.0; 74.0–99.0)
CBR^a^, *n* (%, 95% CI)	16 (64.0; 42.5–82.0)

CBR, clinical benefit rate; CI, confidence interval; DCR, disease control rate; DoR, duration of response; NR, not reported; ORR, objective response rate; PD-L1, programmed death-ligand 1.

a Defined as the proportion of patients with complete or partial response or stable disease for at least 24 weeks.

**Figure 3:**
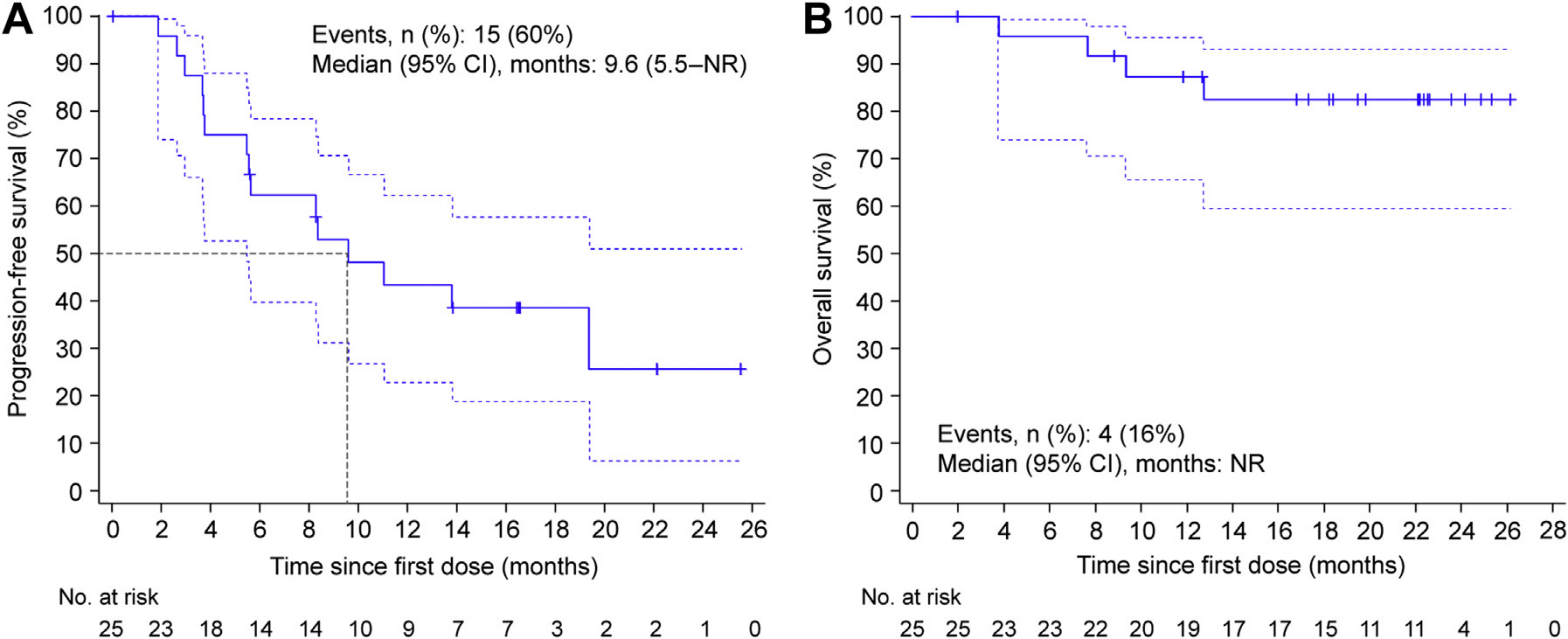
Survival outcomes. Kaplan–Meier curves of progression-free survival (*A*) and overall survival (*B*). The blue dash line shows the 95% CI of the Kaplan–Meier curve. CI, confidence interval; No., number; NR, not reached.

As of the data cutoff, 15 (60.0%) patients had experienced a PFS event and 4 (16.0%) had died. The median PFS was 9.6 months (95% CI: 5.5–not reached; Fig. 3*A*) and the median OS was not reached (Fig. 3*B*). The OS rate at 12 months, 18 months, and 24 months was 87.3% (95% CI: 65.6–95.7), 82.5% (95% CI: 59.6–93.1), and 82.5% (95% CI: 59.6–93.1), respectively. In an exploratory analysis, the median PFS and OS were consistent in patients with PD-L1–positive and PD-L1–negative tumors (Supplementary Fig. 3).

### Safety

The median treatment duration was 11.1 months (interquartile range: 5.1–22.9) with camrelizumab and 11.3 months (interquartile range: 5.7–22.5) with apatinib. All 25 patients experienced at least one treatment-related AE (TRAE) (Table 3). There were 13 patients (52.0%) who required at least one dose reduction in apatinib (Supplementary Table 2). One (4.0%) patient discontinued study treatment because of TRAE (hemoptysis). Grade 3 or higher TRAEs were reported in 20 patients (80.0%), with the most common being increased gamma-glutamyltransferase (six patients [24.0%]), increased alanine aminotransferase (four [16.0%]), and abnormal hepatic function (four [16.0%]). One death (hemoptysis) because of AE was considered treatment-related. Reactive cutaneous capillary endothelial proliferation (RCCEP), a common TRAE associated with camrelizumab, was reported in 13 (52.0%) (grade 1, n = 10; grade 2, n = 2; grade 3, n = 1) patients.A total of nine patients (36.0%) experienced at least one grade 3 or 4 immune-related AE, with the most common being increased aspartate aminotransferase (two patients [8.0%]) (Supplementary Table 3).

**Table 3:** Treatment-Related Adverse Events Occurring in at Least 15% of Patients

Adverse Event	Any Grade	Grade ≥3
Any TRAE	25 (100.0)	20 (80.0)
Alanine aminotransferase increased	15 (60.0)	4 (16.0)
Aspartate aminotransferase increased	14 (56.0)	3 (12.0)
RCCEP	13 (52.0)	1 (4.0)
Hypertension	12 (48.0)	3 (12.0)
Rash	11 (44.0)	0
Proteinuria	11 (44.0)	1 (4.0)
Gamma-glutamyltransferase increased	10 (40.0)	6 (24.0)
Palmar-plantar erythrodysaesthesia syndrome	10 (40.0)	2 (8.0)
Blood bilirubin increased	9 (36.0)	2 (8.0)
Asthenia	9 (36.0)	0
Hepatic function abnormal	8 (32.0)	4 (16.0)
Anemia	8 (32.0)	0
Platelet count decreased	7 (28.0)	1 (4.0)
Hypothyroidism	7 (28.0)	0
Blood alkaline phosphatase increased	6 (24.0)	3 (12.0)
Pyrexia	6 (24.0)	0
Diarrhea	5 (20.0)	0
White blood cell count decreased	4 (16.0)	0
Blood glucose increased	4 (16.0)	1 (4.0)
Blood pressure increased	4 (16.0)	3 (12.0)
Mouth ulceration	4 (16.0)	0
Decreased appetite	4 (16.0)	1 (4.0)
Hemoptysis	4 (16.0)	1 (4.0)
Headache	4 (16.0)	1 (4.0)

*Note:* Data are n (%). All grade 3 or higher TRAEs occurring in more than one patient are listed.

RCCEP, reactive cutaneous capillary endothelial proliferation; TRAE, treatment-related adverse event.

## Discussion

Low-dose angiogenesis inhibitors were previously found to optimize the tumor microenvironment and potentiate the antitumor effect of PD-1 and PD-L1 blockade in lung cancer. In this cohort of phase 2 dose-expansion trial, camrelizumab plus a low dose of apatinib as first-line therapy led to an encouraging ORR of 40% and median PFS of 9.6 months together with long-term survival in patients with high-TMB NSCLC irrespective of tumor PD-L1 expression. More importantly, only 4.0% of patients discontinued study treatment because of TRAE, suggesting that the strategy of low-dose VEGF receptor (VEGFR) TKIs merits further validation in phase 3 trials.

Currently, the established first-line therapy for PD-L1 unselected nonsquamous NSCLC without driver mutation is the immunotherapy-chemotherapy combination. The ORR and DCR reported with first-line pembrolizumab or atezolizumab (with or without bevacizumab) plus chemotherapy was 47% to 63.5% and 79.6% to 85.3%, respectively.^5^^,^^7^^,^^8^^,^^34^ In this study, the ORR and DCR were 40% and 92.0% with the chemo-free regimen of camrelizumab plus apatinib, generally comparable to those achieved with the immunotherapy-chemotherapy combination. More importantly, the benefit of camrelizumab plus apatinib was durable, with a median DoR of 15.6 months. The median PFS of 9.6 months also compared favorably with the 8.8 months for pembrolizumab plus chemotherapy and 7.6 to 8.3 months for atezolizumab-based regimens as first-line treatment for nonsquamous NSCLC.^5^^,^^7^^,^^8^^,^^34^ After a follow-up of 19.5 months, the median OS was not reached yet and the estimated 2-year OS rate was a remarkable 82.5%. The survival curve exhibited a plateau in the right tail, suggesting potential benefits beyond initial treatment. Taken together with the results from the phase 1 study of sintilimab plus anlotinib, by using a strategy of 2 weeks on and then 1 week off,^19^ these data collectively illustrate the potential of the combination of an ICI with a low dose of an antiangiogenic agent for the frontline treatment of advanced NSCLC. However, the LEAP 007 study reported that pembrolizumab with a full dose of lenvatinib failed to prolong the OS (14.1 versus 16.4 mo) even though it improved the ORR and prolonged the PFS when compared with pembrolizumab in patients with advanced NSCLC.^35^ Because TRAE-related discontinuation happened in 27.5% of patients in the lenvatinib group, the possible explanation for the results of the LEAP 007 study might be that the adverse effects of full-dose lenvatinib hindered its benefit of long-time survival. Conversely, only 4.0% of patients discontinued study treatment because of TRAE in this study, suggesting low-dose apatinib plus PD-1 or PD-L1 blockade is worth further validation in phase 3 trials.

TMB has been used as a biomarker for ICI monotherapy or ICI combinations, independent of PD-L1 expression.^18^^,^^33^ In the present study, patients with NSCLC and a high TMB (bTMB ≥1.54 muts/Mb or tTMB >10 muts/Mb) were enrolled, regardless of PD-L1 expression. This cutoff of high TMB was on the basis of the trials of nivolumab plus ipilimumab (tTMB) and our previous study of camrelizumab plus apatinib (bTMB) in patients with NSCLC.^29^^,^^31^^,^^32^^,^^36^ According to these reports, the frequency of patients with high TMB was approximately 50%,^29^^,^^32^^,^^36^ illustrating the relevance of treatment regimen targeting this population. Notably, the cutoff of 1.54 muts/Mb for high bTMB used in our trial was lower than reported in other trials, possibly because of the difference in the method of TMB calculation.^37^^,^^38^ For example, bTMB was calculated as somatic base substitutions with greater than or equal to 0.5% allele frequency in the BFAST study compared with base substitutions plus indels with greater than or equal to 1% allele frequency in our analysis. In the present study, 9.5% of patients failed the trial screen because of low TMB, and subgroup analysis revealed comparable antitumor activity of camrelizumab plus apatinib in high-TMB patients with positive and negative PD-L1 expression, suggesting more efforts are needed to implement TMB or the other potent biomarkers to guide the combination of immunotherapy and VEGFR-TKIs in patients with NSCLC.

The safety profile of the camrelizumab and apatinib combination was generally consistent with that of the individual agents and other combinations of PD-L1 inhibitor plus antiangiogenic agent,^15^, ^16^, ^17^, ^18^^,^^35^ with no new safety signals identified. In this study, the most common grade 3 to 4 TRAEs were abnormalities in hepatic function tests (12%–24%). The incidence of hepatic toxicities reported here with camrelizumab plus apatinib for treatment of nonsquamous NSCLC in the first-line setting was slightly higher than that previously reported for this combination in the second-line or later setting,^29^ probably because of prolonged duration of drug exposure. In addition, grade 3 to 4 hypertension occurred in 12% of patients, consistent with the classic effect of antiangiogenic drugs. Nevertheless, these events were manageable with dose interruptions and modifications (of apatinib) and supportive care, and the incidence of treatment discontinuation because of TRAE was low (4.0%). Hepatic toxicities and hypertension are important risks of combination therapy and require careful monitoring and appropriate intervention. Overall, the most frequent TRAE observed in this study was low-grade RCCEP (52%), a self-resolving AE known to be associated with camrelizumab.^39^ In advanced solid tumors, the incidence of RCCEP reported with camrelizumab monotherapy was 67% to 97%.^24^^,^^27^^,^^40^, ^41^, ^42^ Consistent with our previous study in pretreated NSCLC,^29^ the addition of apatinib to camrelizumab decreased the incidence of RCCEP, suggesting the possible involvement of the VEGFA-VEGFR2 pathway in the pathogenesis of RCCEP.

The major limitation of the study was intrinsic to an early-phase study and included small sample size and lack of a control arm. The efficacy and safety of the combination therapy will need to be confirmed in larger, randomized controlled trials. Second, tumor response was not assessed by an independent review committee in our study. In addition, the screen failure rate was relatively high. Because *EGFR* and *ALK* mutations were detected after the signed informed consent in the study, the high screen failure rate was partially because of the high *EGFR* mutation rate in Asian patients with nonsquamous NSCLC. On the other hand, TMB detection also limited patient recruitment with a prolonged screening period (a major reason for patient withdrawal) and exclusion of patients with low TMB. The performance of low-dose VEGFR-TKI in combination with immunotherapy remains to be confirmed in a broader patient population with NSCLC, including in non-Asian patients.

In conclusion, frontline camrelizumab plus low-dose apatinib exhibited promising clinical activity with acceptable safety in patients with advanced nonsquamous NSCLC and high TMB regardless of PD-L1 expression. The combination presents a potential new treatment option for advanced NSCLC and warrants further validation.

## CRediT Authorship Contribution Statement

**Caicun Zhou, Quanren Wang:** Conceptualization.

**Shengxiang Ren, Jianxing He, Yong Fang, Gongyan Chen, Zhiyong Ma, Jianhua Chen, Renhua Guo, Xiaoyan Lin, Yu Yao, Gang Wu, Caicun Zhou:** Investigation.

**Quanren Wang, Caicun Zhou:** Supervision.

**Shengxiang Ren, Caicun Zhou:** Writing - original draft.

**Shengxiang Ren, Jianxing He, Yong Fang, Gongyan Chen, Zhiyong Ma, Jianhua Chen, Renhua Guo, Xiaoyan Lin, Yu Yao, Gang Wu, Quanren Wang, Caicun Zhou:** Writing - review & editing.
