# Supplementary material for: Camrelizumab Plus Apatinib in Treatment-Naive Patients With Advanced Nonsquamous NSCLC: A Multicenter, Open-Label, Single-Arm, Phase 2 Trial
Source: JTO Clin Res Rep. 2022 Mar 30;3(5):100312. doi: 10.1016/j.jtocrr.2022.100312 (PMC9046448; doi:10.1016/j.jtocrr.2022.100312)
Supplement: Supplementary Tables and Figures [file mmc1.docx]

**Supplementary files**

Table of Contents

[Table S1. Tumor mutational burden of individual patients 2](#_Toc95481843)

[Table S2. Summary of treatment-related adverse events. 3](#_Toc95481844)

[Table S3. Immune-related adverse events. 4](#_Toc95481845)

[Figure S1. Overview of study design. 5](#_Toc95481846)

[Figure S2. Determination of bTMB cutoff for prediction of tumor response. 6](#_Toc95481847)

[Figure S3. Survival outcomes by PD-L1 expression 7](#_Toc95481848)

[Protocol amendments 8](#_Toc95481849)

# Table S1. Tumor mutational burden of individual patients

| Patient No. | tTMB | bTMB |
| --- | --- | --- |
| 1 | 1.03 | 1.54 |
| 2 | 1.54 | 2.05 |
| 3 | 2.56 | 1.54 |
| 4 | 3.59 | 4.10 |
| 5 | 5.13 | 3.08 |
| 6 | 6.15 | 2.56 |
| 7 | NA | 2.56 |
| 8 | NA | 6.15 |
| 9 | NA | 3.59 |
| 10 | NA | 4.62 |
| 11 | NA | 2.05 |
| 12 | NA | 7.18 |
| 13 | NA | 3.08 |
| 14 | NA | 9.74 |
| 15 | NA | 2.05 |
| 16 | NA | 4.10 |
| 17 | NA | 2.05 |
| 18 | NA | 6.15 |
| 19 | NA | 3.59 |
| 20 | NA | 2.05 |
| 21 | NA | 13.33 |
| 22 | NA | 2.05 |
| 23 | NA | 1.54 |
| 24 | NA | 4.10 |
| 25 | NA | 4.62 |
| Median (range),  muts/Mb | 3.08 (1.03-6.15) | 3.08 (1.54-13.33) |

bTMB, blood tumor mutational burden; NA, not applicable; tTMB, tissue tumor mutational burden.

# Table S2. Summary of treatment-related adverse events.

|  | Patients (n = 25) |
| --- | --- |
| Any TRAE | 25 (100.0) |
| Grade ≥3 | 20 (80.0) |
| SAE | 9 (36.0) |
| TRAE leading to discontinuation of any treatment component | 8 (32.0) |
| Discontinuation of camrelizumab | 3 (12.0) |
| Discontinuation of apatinib | 6 (24.0) |
| Discontinuation of camrelizumab and apatinib | 1 (4.0) |
| TRAE leading to dose reduction of apatinib | 13 (52.0) |
| TRAE leading to death | 1 (4.0) |

Data are n (%). SAE, serious adverse event; TRAE, treatment-related adverse event.

# Table S3. Immune-related adverse events.

|  | **Any grade** | **Grade ≥3** |
| --- | --- | --- |
| Hypothyroidism | 6 (24.0) | 0 |
| Rash | 6 (24.0) | 0 |
| Proteinuria | 4 (16.0) | 1 (4.0) |
| Hepatic function abnormal | 3 (12.0) | 1 (4.0) |
| Asthenia | 4 (16.0) | 0 |
| Pyrexia | 3 (12.0) | 0 |
| Diarrhoea | 3 (12.0) | 0 |
| Aspartate aminotransferase increased | 2 (8.0) | 2 (8.0) |
| Gamma-glutamyltransferase increased | 2 (8.0) | 1 (4.0) |
| Blood alkaline phosphatase increased | 2 (8.0) | 1 (4.0) |
| Mouth ulceration | 2 (8.0) | 0 |
| Decreased appetite | 2 (8.0) | 0 |
| Alanine aminotransferase increased | 2 (8.0) | 0 |
| Blood bilirubin increased | 1 (4.0) | 1 (4.0) |
| Pneumonitis | 1 (4.0) | 1 (4.0) |
| Diabetes mellitus | 1 (4.0) | 1 (4.0) |
| Type 2 diabetes mellitus | 1 (4.0) | 1 (4.0) |
| Hypophysitis | 1 (4.0) | 0 |
| Hyperthyroidism | 1 (4.0) | 0 |
| Immune-mediated hypophysitis | 1 (4.0) | 0 |
| Abdominal pain | 1 (4.0) | 0 |
| Dry mouth | 1 (4.0) | 0 |
| Gastritis | 1 (4.0) | 0 |
| Hyperglycaemia | 1 (4.0) | 0 |
| Immune-mediated hepatitis | 1 (4.0) | 0 |
| Interstitial lung disease | 1 (4.0) | 0 |
| Immune-mediated lung disease | 1 (4.0) | 0 |
| Nasopharyngitis | 1 (4.0) | 0 |
| Myalgia | 1 (4.0) | 0 |
| Pyogenic granuloma | 1 (4.0) | 0 |

Data are n (%).


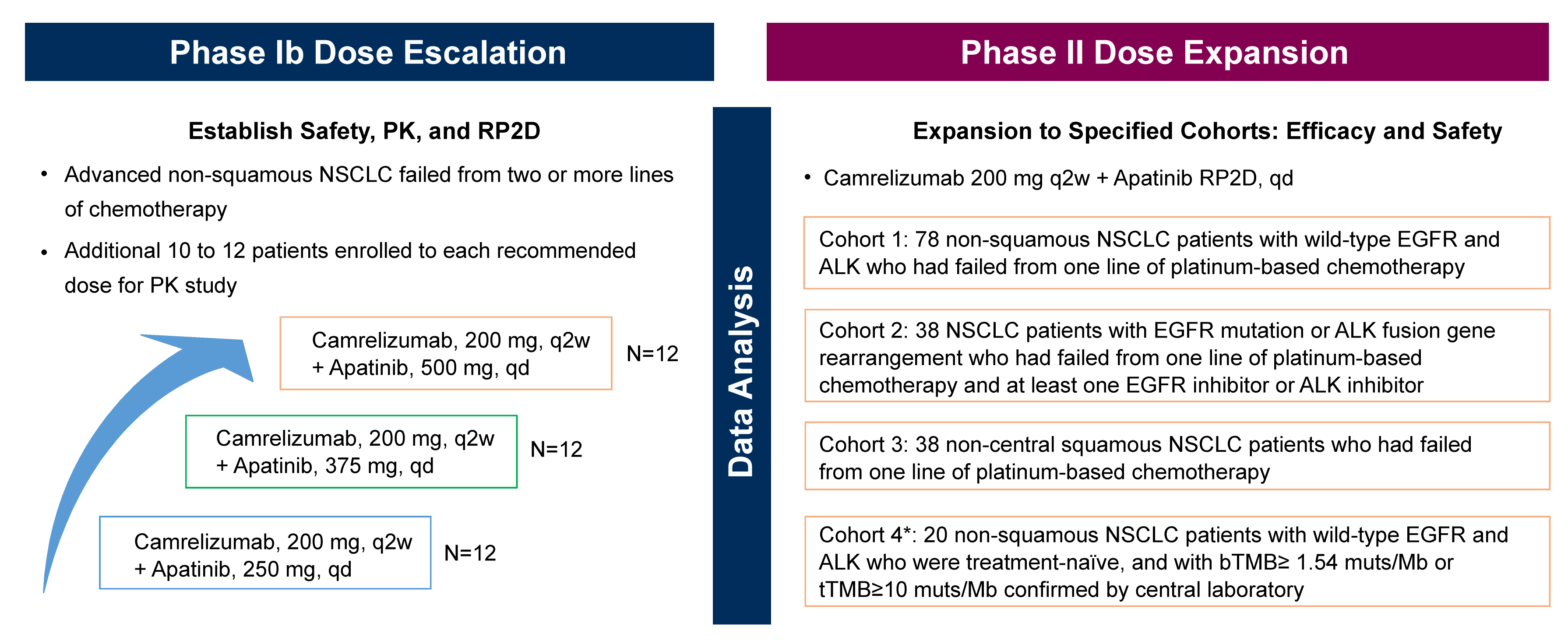


Figure S1. Overview of study design. * Phase II Cohort 4 was added to the protocol after data analysis of cohort 1. PK, pharmacokinetics; RP2D, recommended phase 2 dose.


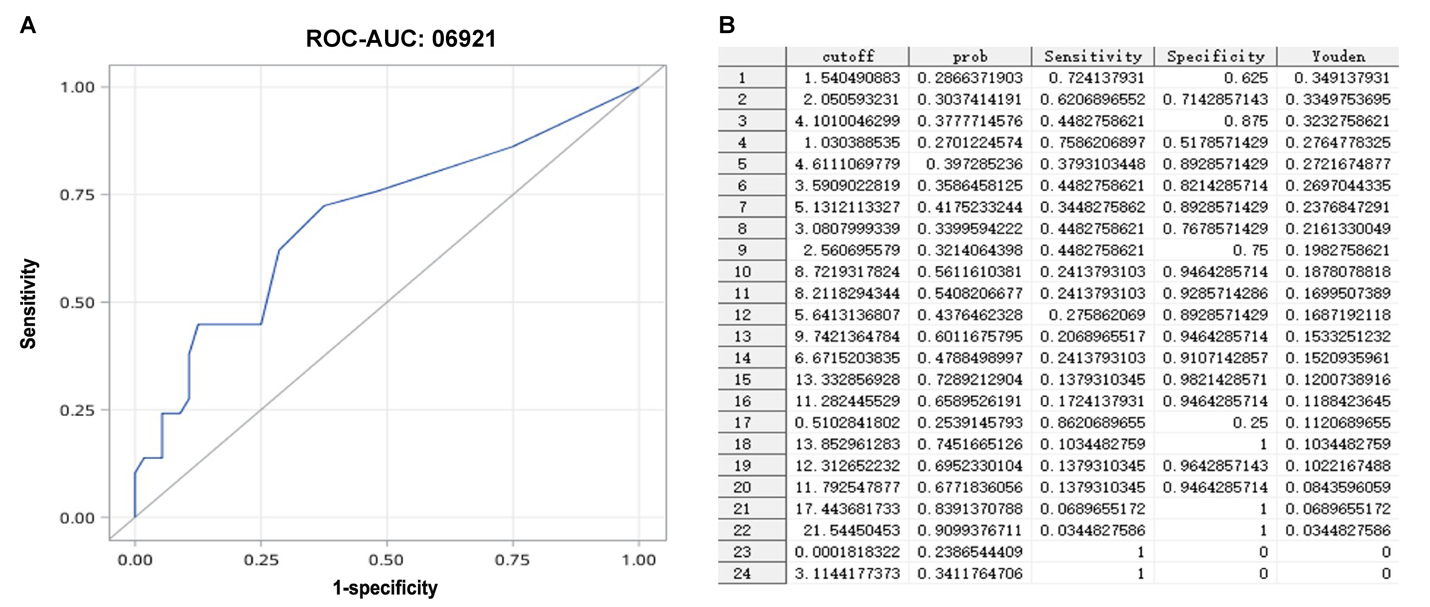


Figure S2. Determination of bTMB cutoff for prediction of tumor response. In cohort 1 of the study, bTMB was evaluable in 80 patients with non-small-cell lung carcinoma and was assessed using the same method as described in this paper. The relationship between bTMB and tumor response was explored using receiver operating characteristic (ROC) analysis and an area under the ROC curve of 0.6921 was achieved (Panel A). Based on the Youden index, a bTMB cutoff of 1.54 mut/Mb provided the best discrimination of treatment response (sensitivity, 72.4%; specificity, 62.5%; Panel B). The objective response rate was 50.0% (19/38) in patients with bTMB ≥1.54 mut/Mb, and 16.7% (7/42) in those with bTMB <1.54 mut/Mb. bTMB, blood tumor mutational burden.


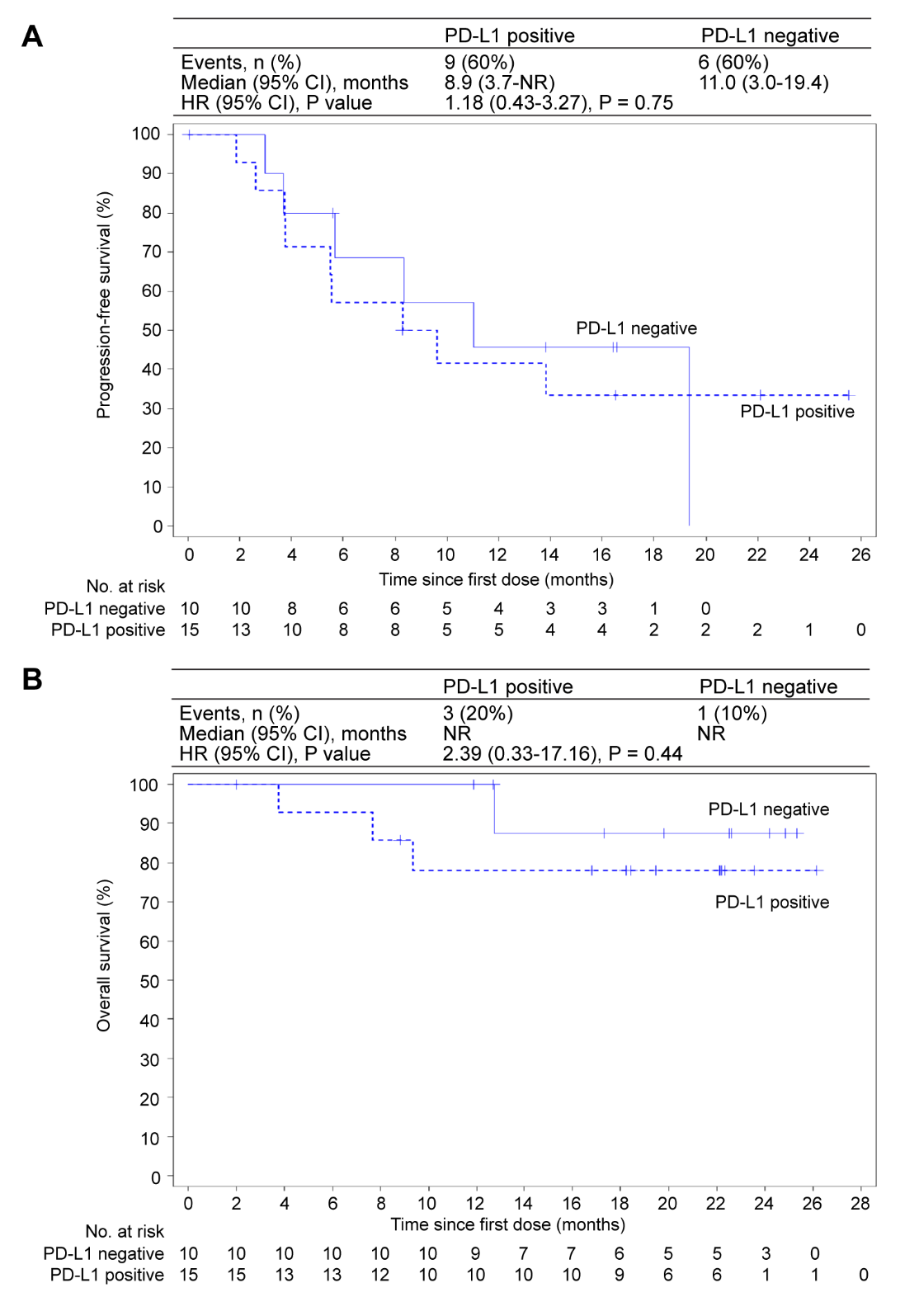


Figure S3. Survival outcomes by PD-L1 expression. Kaplan-Meier curves of progression-free survival (A) and overall survival (B). PD-L1-positive was defined as a tumor proportion score of ≥1%. HR and the 95% CIs were estimated using the COX proportional hazards model. HR, hazard ratio; NR, not reached.

# Protocol amendments

| Section | Protocol  Version Date: Jul 1, 2018  Version: 4.0 | Protocol  Version Date: Dec 10, 2018  Version: 5.0 |
| --- | --- | --- |
| Inclusion Criteria | 4. Phase II: Patients who have undergone only one previous platinum-based two-drug chemotherapy regimen but failed the treatment.  …  According to the type of driver gene mutations and pathological classification, the subjects are divided into the following three cohorts:  … | 4. Phase II: Patients who have undergone only one previous platinum-based two-drug chemotherapy regimen but failed the treatment or showed recurrence, or patients in the stage of palliative treatment who have received no systemic treatment (only for cohort 4).  …  According to the type of driver gene mutations and pathological classification, the subjects are divided into the following four cohorts:  …  Cohort 4. Non-squamous and non-small cell lung cancer with wild-type EGFR and ALK and with bTMB ≥1.54 muts/Mb or tTMB >10 muts/Mb confirmed by central laboratory, and patients in the stage of palliative treatment who have received no systemic treatment. |
| Determination of Sample Size | There are three cohorts for Phase II.  …  Total 154 subjects are required for phase II. | There are four cohorts for Phase II.  …  For cohort 4. Assuming an objective response rate is 50% and the width of the 90% confidence interval is 0.4, 20 subjects are required to be enrolled. Total 174 subjects are required for phase II. |
| Trial Flow Chart | / | [28] For cohort 4, the test results of tumor mutation burden (TMB) in the peripheral blood and/or tumor tissues will be verified before the subjects take the first dose of the investigational product, so the TMB test for cohort 4 must be completed before the first dose. However, the delay in the screening period caused by TMB testing is allowed, but other tests (including imaging examinations) must be completed within 21 days prior to the first dose. |
